# Cook County Hospital

**Published:** 1875-09

**Authors:** 


### Hospitals.

### COOK COUNTY HOSPITAL.

*Case of Pyonephrosis*— (From Notes by D. A. K. STEELE, M.D.)

Thos. M., aged 18 ; laborer ; Bohemian by birth. Was admitted to wards of the County Hospital, June 20, 1874, suffering from obscure renal difficulty. As he spoke English very imperfectly, the previous history of the case was very meagre, and with much difficulty the following history was elicited :

He had not been feeling well for three months ; had some difficulty in urinating ; complained of occasional pains in the regions of the kidneys, also of cramping pains in the legs. For the past nine days says he has been much worse ; pains aggravated ; has had occasional rigors ; breath short and labored ; suffered from occasional convulsions. On admission, patient fairly nourished ; skin hot ; pulse, 120° ; temp., 100° ; resp., 36 ; tongue somewhat furred ; face dusky and anxious.

Physical examination reveals lung sounds normal ; a soft, blowing murmur at base of heart with first sound ; abdomen seems tender on palpation, especially at the sides ; has some diarrhœa ; makes water frequently, but passes only a small quantity of high-colored urine, which contains a considerable sediment, has a sp. gr. 10.10, contains pus and a large percentage of albumen. Patient was put upon 20 gtt. doses of digitalis and 5 grs. pot. iodid. ter die, and sufficient morphia to allay pain ; to have light, nutritious diet.

June 24th. Patient continued nearly in the same condition, respiration becoming more labored, pyrexia more marked ; occasionally the patient becomes somewhat delirious.

25th. Patient more delirious ; attempted to escape from the hospital last night, and was with difficulty overcome and brought back.

2 P. M. Patient rapidly growing worse; breathing more labored; expression of the face more anxious; eyes wild in expression, pupils dilated; has occasional tetanic convulsions. Died at 2.45 P. M., after a very severe convulsion. Autopsy, eighteen hours after death.

On examination of the brain, found membranes apparently normal; pia mater was partially covered with cloudy serum; found considerable serous effusion about base of brain; brain substance slightly congested, the puncta being very prominent. On opening thorax, found left lung healthy, the right bound down at the apex by old pleuritic adhesions, does not contain any tubercles; heart, normal; liver somewhat congested. On removing the left kidney found it enlarged and lobulated, fluctuating freely, being filled with pus which had so completely distended the pelvis that the glandular structure of the organ was entirely disorganized, leaving only a large membranous sac which was subdivided into several compartments, giving it a multilocular appearance, there being free communication between all portions. Also, found five calculi occupying the position of the calyces, principally occupying the portion around the opening of the ureter, the largest calculus weighing 27½ grains; the ureter was somewhat dilated.

The right kidney was similarly enlarged, and full of pus and urine mixed. Its substance was not so completely disorganized as the left. It also contained four or five calculi, the largest weighing 18 grains and perfectly occluding the opening of the ureter. The right ureter was also dilated. Found the walls of the bladder nearly twice as thick as they should be, and in a state of chronic inflammation. It contained a considerable quantity of turbid urine. Other abdominal organs were healthy. The entire weight of the calculi of both kidneys was fifty-seven grains.

